# DOES THE FACTS ON AGING QUIZ ASSESS AGING STEREOTYPES?

**DOI:** 10.1093/geroni/igad104.2702

**Published:** 2023-12-21

**Authors:** Savannah Schultz, Sarah Francis, Mack Shelley, Aaron Vincent, Cori Hyde, Alexandra Bauman

**Affiliations:** Iowa State University, Ames, Iowa, United States; Iowa State University, Ames, Iowa, United States; Iowa State University, Ames, Iowa, United States; Iowa State University, Ames, Iowa, United States; Iowa State University, Ames, Iowa, United States; Iowa Department on Aging, National Resource Center on Nutrition & Aging, Des Moines, Iowa, United States

## Abstract

The World Health Organization (WHO) Global Report on Ageism urges researchers to develop and validate assessment tools, thus enabling the field to better address ageism. This study assesses the internal consistency and construct validity of a short Facts on Aging Quiz (SFAQ, 10-items). We hypothesize that the SFAQ is a valid and reliable tool for assessing aging stereotypes. Data from the National Resource Center on Nutrition and Aging 2022 Needs Assessment were utilized. Data were collected via an online survey through a convenience sample of the National Aging Network (n=1,910). Three aging perspective tools were included: SFAQ, Expectations Regarding Aging (ERA, 12-items), and the WHO Ageism Quiz (AQ, 8-items). An exploratory factor analysis assessed whether negative (NEG) and positive (POS)-stereotype SFAQ items emerged as constructs. The factor analysis showed that NEG and POS-SFAQ accounted for 31.39% and 19.63% of the total variance, respectively. Cronbach alpha evaluated the internal consistency of the tools. Internal consistency for ERA (α=.81) and AQ (α=.57) were acceptable but low for total SFAQ (α=.28); reliability was acceptable for NEG-SFAQ (α=.71) and lower for POS-SFAQ (α=.40). Pearson correlation assessed convergent validity, with a significance level of p<.0001 (df=1,908) for all correlations. A negative correlation was detected between POS-SFAQ and ERA (r=-.31) and positive correlations between NEG-SFAQ and both ERA (r=.55) and AQ (r=.65), confirming convergent validity. POS-SFAQ was negatively correlated with NEG-SFAQ (r=-.19), suggesting the SFAQ may contain conflicting items. In conclusion, versions of the FAQ may perform better as a tool to assess aging stereotypes.

